# Predicting left ventricular hypertrophy from the 12-lead electrocardiogram in the UK Biobank imaging study using machine learning

**DOI:** 10.1093/ehjdh/ztad037

**Published:** 2023-06-01

**Authors:** Hafiz Naderi, Julia Ramírez, Stefan van Duijvenboden, Esmeralda Ruiz Pujadas, Nay Aung, Lin Wang, Choudhary Anwar Ahmed Chahal, Karim Lekadir, Steffen E Petersen, Patricia B Munroe

**Affiliations:** William Harvey Research Institute, NIHR Barts Biomedical Research Centre, Queen Mary University of London, Charterhouse Square, London, EC1M 6BQ, UK; Barts Heart Centre, St Bartholomew’s Hospital, Barts Health NHS Trust, West Smithfield, London, EC1A 7BE, UK; William Harvey Research Institute, NIHR Barts Biomedical Research Centre, Queen Mary University of London, Charterhouse Square, London, EC1M 6BQ, UK; Aragon Institute of Engineering Research, University of Zaragoza, Zaragoza, Spain; William Harvey Research Institute, NIHR Barts Biomedical Research Centre, Queen Mary University of London, Charterhouse Square, London, EC1M 6BQ, UK; Big Data Institute, La Ka Shing Centre for Health Information and Discovery, University of Oxford, Oxford, UK; Faculty of Mathematics and Computer Science, University of Barcelona, Barcelona, Spain; William Harvey Research Institute, NIHR Barts Biomedical Research Centre, Queen Mary University of London, Charterhouse Square, London, EC1M 6BQ, UK; National Institute of Health and Care Research Barts Biomedical Research Centre, Queen Mary University of London, Charterhouse Square, London, EC1M 6BQ, UK; Barts Heart Centre, St Bartholomew’s Hospital, Barts Health NHS Trust, West Smithfield, London, EC1A 7BE, UK; School of Electronic Engineering and Computer Science, Queen Mary University of London, London, UK; Barts Heart Centre, St Bartholomew’s Hospital, Barts Health NHS Trust, West Smithfield, London, EC1A 7BE, UK; Cardiac Electrophysiology Section, Division of Cardiovascular Diseases, University of Pennsylvania, Philadelphia, PA, USA; Department of Cardiovascular Diseases, Mayo Clinic, Rochester, MN, USA; Faculty of Mathematics and Computer Science, University of Barcelona, Barcelona, Spain; William Harvey Research Institute, NIHR Barts Biomedical Research Centre, Queen Mary University of London, Charterhouse Square, London, EC1M 6BQ, UK; National Institute of Health and Care Research Barts Biomedical Research Centre, Queen Mary University of London, Charterhouse Square, London, EC1M 6BQ, UK; Barts Heart Centre, St Bartholomew’s Hospital, Barts Health NHS Trust, West Smithfield, London, EC1A 7BE, UK; Health Data Research UK, Gibbs Building, 215 Euston Road, London, NW1 2BE, UK; Alan Turing Institute, The British Library, 96 Euston Road, London, NW1 2DB, UK; William Harvey Research Institute, NIHR Barts Biomedical Research Centre, Queen Mary University of London, Charterhouse Square, London, EC1M 6BQ, UK; National Institute of Health and Care Research Barts Biomedical Research Centre, Queen Mary University of London, Charterhouse Square, London, EC1M 6BQ, UK

**Keywords:** Left ventricular hypertrophy, Electrocardiogram, Cardiovascular magnetic resonance imaging, Machine learning, Cardiovascular screening

## Abstract

**Aims:**

Left ventricular hypertrophy (LVH) is an established, independent predictor of cardiovascular disease. Indices derived from the electrocardiogram (ECG) have been used to infer the presence of LVH with limited sensitivity. This study aimed to classify LVH defined by cardiovascular magnetic resonance (CMR) imaging using the 12-lead ECG for cost-effective patient stratification.

**Methods and results:**

We extracted ECG biomarkers with a known physiological association with LVH from the 12-lead ECG of 37 534 participants in the UK Biobank imaging study. Classification models integrating ECG biomarkers and clinical variables were built using logistic regression, support vector machine (SVM) and random forest (RF). The dataset was split into 80% training and 20% test sets for performance evaluation. Ten-fold cross validation was applied with further validation testing performed by separating data based on UK Biobank imaging centres. QRS amplitude and blood pressure (*P* < 0.001) were the features most strongly associated with LVH. Classification with logistic regression had an accuracy of 81% [sensitivity 70%, specificity 81%, Area under the receiver operator curve (AUC) 0.86], SVM 81% accuracy (sensitivity 72%, specificity 81%, AUC 0.85) and RF 72% accuracy (sensitivity 74%, specificity 72%, AUC 0.83). ECG biomarkers enhanced model performance of all classifiers, compared to using clinical variables alone. Validation testing by UK Biobank imaging centres demonstrated robustness of our models.

**Conclusion:**

A combination of ECG biomarkers and clinical variables were able to predict LVH defined by CMR. Our findings provide support for the ECG as an inexpensive screening tool to risk stratify patients with LVH as a prelude to advanced imaging.

## Graphical Abstract

**Graphical Abstract:**
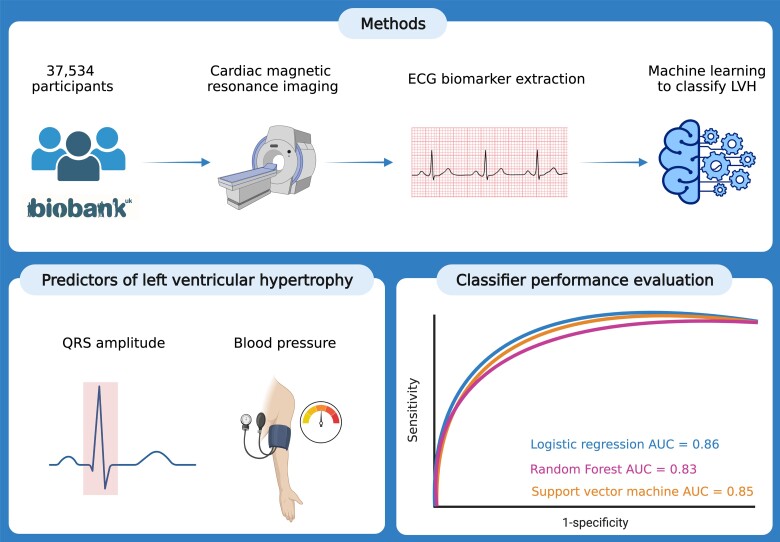
Created using BioRender.com. AUC: area under the receiver operator curve, LVH: left ventricular hypertrophy.

## Introduction

Left ventricular hypertrophy (LVH) is pathologically increased LV mass and an established, independent predictor of cardiovascular morbidity and mortality.^1–4^ Two-dimensional echocardiography is used for the evaluation of LV mass, however, it remains operator dependent and poor acoustic windows limit its use.^5^ Cardiovascular magnetic resonance (CMR) imaging is considered the gold standard imaging modality in the assessment of LVH as it is accurate, reproducible and non-invasive. CMR imaging enables comprehensive assessment of LVH by obtaining precise measurements of chamber size and advanced techniques such as late gadolinium enhancement. In addition, parametric mapping, diffusion tensor imaging and myocardial strain can help to differentiate key aetiologies of LVH for prognostication.^6^ Individuals with CMR evidence of LVH are at greater risk of cardiovascular events compared with normal LV geometry.^7^ However, CMR is limited in our healthcare system due to cost and availability, therefore a cost-effective approach would be beneficial to identify individuals with LVH.

In contrast to CMR imaging, the electrocardiogram (ECG) is an inexpensive screening tool to detect LVH at the bedside, ubiquitous and technically easy to perform. Despite its high specificity for LVH detection, studies have consistently highlighted its limited sensitivity, ranging 15–30%.^8,9^ To address this, recent work has demonstrated LVH detection using deep learning on 12-lead ECG, showing correlation with CMR-derived LV mass.^10–12^ However, a study comparing supervised machine learning techniques using a combination of selected ECG biomarkers to classify LVH is lacking. Deep learning algorithms use agnostic approaches for LVH detection and don’t allow the identification of specific ECG biomarkers contributing to LVH for potential mechanistic insights.

This study aims to assess the discriminative potential of a combination of automatically extracted ECG biomarkers, together with clinical variables, to optimally classify LVH defined by CMR imaging in a large community population using supervised machine learning techniques. Our overall goal is to explore the potential of the ECG to be used as a screening tool for LVH detection, offering a cost-effective approach identify LVH for risk stratification as a prelude to advanced imaging.

## Methods

### Study population

The UK Biobank is a large prospective population study where demographics, medication history, electronic health records, biomarkers and genomics were collected in half a million participants aged 40–69 years when recruited between 2006 and 2010 from across the United Kingdom. The UK Biobank imaging study was launched in 2015, with the aim of scanning 20% of the original cohort, that is 100 000 participants.^13^ The details of the UK Biobank CMR protocol have been described elsewhere.^14^

**Figure 1:**
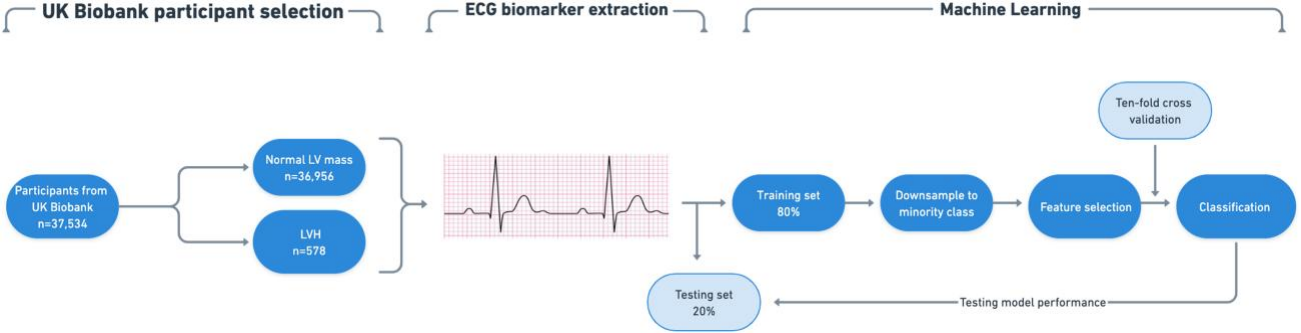
Flow diagram illustrating the steps involved in UK Biobank participant selection, ECG biomarker extraction and machine learning. Abbreviations: ECG: electrocardiogram, LV: left ventricle, LVH: left ventricular hypertrophy.

A total of 44 817 participants had completed the UK Biobank imaging study. Accounting for incomplete CMR and ECG data, a total of 37 534 participants were categorised into normal LV and LVH using CMR parameters, which have been derived using a fully automated quality-controlled image analysis pipeline previously developed and validated in a large subset of the UK Biobank.^15,16^ LVH was defined as indexed LV mass >70 g/m^2^ (men) and >55 g/m^2^ (women) with respect to normal ranges published in the group.^15^ Indexing for body surface area was performed using the Mosteller formula.^17^ The proportion of UK Biobank participants in each category is shown in *Table 1*. *Figure 1* illustrates the sample selection process and subsequent steps in the methodology.

**Table 1:** UK Biobank participant characteristics

	Overall	Normal LV mass	LVH	*P*-value
	(*n* = 37 534)	(*n* = 36 956)	(*n* = 578)	
Age (years)	64 [58, 70]	64 [58, 70]	64 [57, 70]	0.1
Sex (%)				0.1
Female	19 529 (52.0)	19 252 (52.1)	277 (47.9)	
BMI (kg/m^2^)	26.0 [23.6, 28.8]	26.0 [23.6, 28.7]	26.2 [24.0, 29.7]	**0**.**01**
Ethnicity (%)				0.6
White European	36 342 (96.8)	35 784 (96.8)	558 (96.5)	
Other	1192 (3.2)	1172 (3.2)	20 (3.5)	
Potential causes of LVH				
Hypertension (%)	27 946 (74.5)	27 431 (74.2)	515 (89.1)	**<0**.**001**
Hypertrophic cardiomyopathy (%)	4034 (10.7)	3967 (10.7)	67 (11.6)	**0**.**6**
Phenocopies	31 (0.1)	30 (0.1)	1 (0.1)	**0**.**4**
Systolic BP (mmHg)	141 [128, 157]	141 [128, 156]	156 [142, 174]	**<0**.**001**
Diastolic BP (mmHg)	81 [74, 89]	81[74, 89]	87 [77, 96]	**<0**.**001**
High cholesterol (%)	18 981 (50.4)	22 705 (61.4)	343 (59.3)	0.3
Total cholesterol (mmol/L)	4.6 [4.0, 5.2]	4.6 [4.0, 5.2]	4.6 [4.0, 5.2]	0.5
Non-HDL cholesterol (mmol/L)	3.2 [2.7, 3.8]	3.2 [2.7, 3.8]	3.3 [2.7, 3.8]	0.9
Medication use				
Anti-hypertensive medication (%)	9208 (24.5)	8982 (24.3)	226 (39.1)	**<0**.**001**
Lipid lowering medication (%)	9454 (25.2)	9302 (25.2)	152 (26.3)	0.5
Diabetes (%)	2064 (5.5)	2016 (5.5)	48 (8.3%)	**0**.**006**
Smoking status (%)				**0**.**01**
Never	22 737 (60.6)	22 408 (60.6)	329 (56.9)	
Previous	12 450 (33.2)	12 254 (33.2)	196 (33.9)	
Current	2347 (6.3)	2294 (6.2)	53 (9.2)	
Alcohol intake (%)				0.3
Never	1731 (4.6)	1710 (4.6)	21 (3.6)	
Current	35 803 (95.4)	35 246 (95.4)	557 (96.4)	
Global ECG indices				
Sokolow–Lyon (%)	595 (1.6)	558 (1.5)	37 (6.4)	**<0**.**001**
Cornell Voltage (%)	2689 (7.2)	2644 (7.2)	45 (7.8)	0.6
Pathological Q waves (%)	693 (1.8)	665 (1.8)	28 (4.8)	**<0**.**001**
ST segment deviation (mV)	0.012 [0.002, 0.030]	0.013 [0.002, 0.030]	0.010 [0.001, 0.024]	**<0**.**001**
QT dispersion (ms)	56.9 [37.1, 84.2]	56.6 [37.0, 84.0]	71.7 [48.7, 96.5]	**<0**.**001**
Corrected QT duration (ms)	384.0 [369.2, 384.9]	384.0 [369.2, 399.2]	388.0 [370.5, 405.4]	**<0**.**001**
Positive deflection P wave amplitude (mV)	0.045 [0.026, 0.070]	0.045 [0.026, 0.070]	0.065 [0.034, 0.091]	**<0**.**001**
Positive deflection *P* duration (ms)	52.0 [42.0, 68.0]	52.0 [42.0, 68.0]	48.0 [38.0, 62.0]	**<0**.**001**
Negative terminal *P* amplitude (mV)	-0.038 [-0.061, -0.017]	-0.038 [-0.061, -0.017]	-0.036 [-0.064, -0.012]	0.4
Negative terminal *P* duration (ms)	56.0 [46.0, 66.0]	56.0 [46.0, 66.0]	60.0 [48.0, 70.0]	**<0**.**001**
P wave terminal force in V1 (mV/ms)	-2.34 [-3.62, -0.80]	-2.04 [-3.62, -0.80]	-2.05 [-3.88, -0.61]	0.1
P wave duration (ms)	112.0 [100.0, 124.0]	112.0 [100.0, 124.0]	112.0 [98.0, 122.0]	0.1
Q wave amplitude (mV)	-0.08 [-0.10, -0.06]	-0.08 [-0.10, -0.06]	-0.09 [-0.12, -0.06]	**<0**.**001**
Q wave duration (ms)	23.0 [21.0, 25.0]	23.0 [21.0, 25.0]	24.0 [22.0, 27.0]	**<0**.**001**
R wave amplitude (mV)	0.45 [0.37, 0.60]	0.48 [0.37, 0.60]	0.57 [0.41, 0.71]	**<0**.**001**
S wave amplitude (mV)	-0.29 [-0.39, -0.21]	-0.29 [-0.39, -0.21]	-0.39 [-0.54, -0.28]	**<0**.**001**
QRS amplitude (mV)	0.90 [0.76, 1.07]	0.91 [0.76, 1.06]	1.14 [0.94, 2.82]	**<0**.**001**
QRS duration (ms)	89.0 [82.0, 97.0]	89.0 [81.0, 97.0]	97.0 [89.0, 105.0]	**<0**.**001**
QRS ascending slope (mV/s)	34.2 [27.1, 42.6]	34.2 [27.1, 42.5]	37.1 [29.0, 47.5]	**<0**.**001**
QRS descending slope (mV/s)	-53.4 [-63.4, -54.6]	-53.3 [-63.2, -44.4]	-64.7 [-75.5, -52.3]	**<0**.**001**
T wave amplitude (mV)	0.15 [0.11, 0.19]	0.15 [0.11, 0.19]	0.14 [0.10, 0.18]	**<0**.**001**
T wave duration (ms)	106.0 [100.0, 114.0]	106.0 [100.0, 114.0]	110.0 [102.0, 120.0]	**<0**.**001**
Ventricular rate (beats/min)	61 [56, 68]	62 [56, 68]	58 [52, 64]	**<0**.**001**

Counts variables are presented as number (percentage), continuous variables as median [interquartile range]. To assess for associations between participants with LVH and normal LV mass, the Wilcoxon signed-rank test was used for continuous data and Fisher’s exact test for categorical data. Global ECG indices are the median values calculated from the independent leads of the 12-lead ECG. Blood pressure and cholesterol values are adjusted for medication use. Hypertrophic cardiomyopathy and phenocopies (see Methods for further details). BMI: body mass index, BP: blood pressure, LV: left ventricle, LVH: left ventricular hypertrophy, mmHg: millimetres mercury, mmol/L: millimoles per litre, ms: milliseconds, mV: millivolts, s: seconds.

### ECG biomarker extraction

In the UK Biobank imaging study, participants underwent acquisition of both 12-lead ECG and CMR imaging during the same assessment visit. We analysed the raw 15 s 12-lead ECG signals of each of the 37 534 participants using MATLAB version 2021a to derive biomarkers with a known physiological association with LVH.^18^ A total of 23 ECG biomarkers were extracted (*Table 1*) and only the independent ECG leads (I, II, V1–6) were analysed. Butterworth filter (1–45 Hz) was applied to attenuate baseline wander and high frequency noise. Following R wave detection, signal-averaging of the ECG waveform was derived for each lead and each participant by calculating the median of the available heartbeat waveforms with the same morphology. The Hilbert’s envelope method was used to identify QRS onset and QRS offset (see Supplementary material online, *Figure S1*).^19^ Marker location was obtained from the envelope by taking the tangent from the first derivative before (QRS onset) and after (QRS offset) the R-peak to the isoelectric baseline. Starting at QRS onset, the algorithm finds the points at which the ECG signal crosses the baseline within each complex and labels accordingly. Amplitudes of significant waves within the QRS complex were measured with respect to the QRS onset. There were no participants who had ventricular pacing and no participants were excluded based on bundle branch morphology.

### Calculation of interval-based ECG indices

We also sought to include interval-based ECG indices: QT duration, P wave amplitude, P wave duration, P wave terminal force in V1, T wave duration, and ventricular rate. Classical LVH indices such as Sokolow–Lyon, Cornell voltage, and QT dispersion were calculated from the ECG biomarkers extracted.^20,21^ The tangent method was used to identify T wave end as the tangent from the minimum of the first derivative of the T-wave slope, to the isoelectric line.^22^ Definitions of all ECG biomarkers used in the model can be found in the supplementary material. ECG biomarkers from each independent lead were treated as individual features. In addition, global ECG features were calculated as the median value across the independent leads.

### Ascertainment of clinical variables

In addition to including ECG biomarkers, we also sought to include clinical variables known to be associated with LVH in the classification models (*Table 1*). Each clinical variable was defined by either self-reported questionnaire at the initial assessment visit or biochemistry results. Participants with serum total cholesterol of ≥5 mmol/L and Haemoglobin A1c (HbA1c) ≥ 48 mmol/mol at the baseline visit were considered to have hypercholesterolaemia and diabetes mellitus, respectively. Hypertension was defined according to the ‘high normal’ blood pressure (BP) grade of ≥130/85 mmHg from the latest European Society of Cardiology/European Society of Hypertension guidelines to reflect the demographic of the UK Biobank population.^23^ BP measurements were averaged from two readings taken at the imaging visit. After calculating the average BP values, we adjusted for medication use by adding 15 and 10 mmHg to systolic and diastolic BP, respectively, for participants reported to be taking BP-lowering medication.^24^ We corrected total and non-HDL cholesterol values for participants on cholesterol lowering medication by dividing the total cholesterol by 0.73 and non-HDL cholesterol by 0.66.^25^ The presence of tobacco use was ascertained using self-reported questionnaires at the initial assessment visit, with smoking status classified categorically as current, previous or never. Similarly, alcohol consumption was classified as current or never. To ascertain the approximate number of individuals with hypertrophic cardiomyopathy in our dataset, we reviewed exome sequence data for eight genes implicated in hypertrophic cardiomyopathy.^26^ We report the number of individuals who have rare coding variants with a minor allele frequency of <0.00004 in these genes.^26^ Individuals with potential phenocopies (Fabry disease, amyloidosis, glycogen storage diseases, and RSAopathies) are indicated.^27^

### Supervised machine learning techniques

In order to perform classification, several representative features were extracted from the signal to compose a feature vector (see Supplementary material online, *Table S1*). A selection of three supervised machine learning algorithms were used for classification: logistic regression, support vector machine (SVM) and random forest (RF). The algorithms were implemented in MATLAB and the fit multiclass models for SVMs or other classifiers (fitcecoc) function was used to build the logistic regression and SVM classifiers.^28^ The fit ensemble of learners for classification (fitcensemble) was used to build the RF classifier.^29^ In our experiments, the dataset was split into a training set (80%) for learning and a testing set (20%) for performance evaluation. The parameters we used to assess classifier performance included: accuracy, sensitivity, specificity, and area under the receiver operator curve (AUC). In addition, we applied 10-fold cross validation to the training set. To suitably train the models, all features were standardised using *z*-score standardisation to eliminate scale differences during subsequent classification.

For the SVM classifier, Gaussian kernel function was applied to deal with potential non-linear data.^30^ This transforms a two-dimensional dataset onto a new higher feature space where the classes become separable. For the RF classifier, a number of key parameters were thoroughly optimised in the training set, including the maximal number of branches, as well as the number of features used to split each new node. We also applied bootstrap aggregating, referred to as ‘bagging’, which is a method for generating multiple versions of a predictor and using these to get an aggregated predictor.^31^ The multiple versions of the classification models are formed by making bootstrap replicates of the training set and using these as new training sets. This approach reduces variance and helps avoid overfitting.

### Validation by UK Biobank imaging centre

In addition to applying 10-fold cross validation to the training set, we further assessed robustness of our findings by performing validation according to UK Biobank imaging centres located at different geographical regions. There were four imaging centres that participated in the initial UK Biobank imaging visit located in Cheadle, Newcastle, Reading, and Bristol (*Table 3*). Three sets of validation experiments were performed with rotating training and test sets using different combinations of the imaging centres. This approach was used as we did not currently have access to external datasets for validation.

### Statistical analysis

Statistical analysis was performed using R version 4.0.3 and RStudio Version 1.3.1093.^32^ After excluding missing or extreme outlying ECG values (outside the range defined by the quartiles +/- 1.5× interquartile range) the Classification And REgression Training (CARET) package in R was used for correlation analysis and highly correlated ECG biomarkers were omitted (correlation coefficient threshold of +/- 0.9).^33^ ECG biomarkers with less than 10% of missing data were imputed using the Multivariate Imputation by Chained Equations package in R.^34^ In order to address the imbalance in the dataset, down-sampling was applied using the CARET package in the training set to match the proportion of participants in the minority LVH group. Chi-square test was used to rank the features in terms of feature importance score. To assess for associations, the Wilcoxon signed-rank test was used for continuous data and Fisher’s exact test for categorical data. Normality of continuous data was assessed by visual inspection of histograms and confirmed by the Shapiro–Wilk test. For all analyses, a two-tailed *P* < 0.05 was deemed statistically significant. We included all UK Biobank participants with quality-controlled CMR data available.

## Results

### Study population

*Table 1* summarises clinical and ECG characteristics of the total cohort, normal LV mass and LVH subgroups defined by CMR imaging. Overall, the cohort had a median age of 64 [58, 70] years old and 52% (*n* = 19 529) were female. The proportion of participants with hypertension, high cholesterol, diabetes, and smoking was 74.5%, 50.4%, 5.5%, and 6.3%, respectively. In the total cohort the frequency of participants with criteria for Sokolow–Lyon, Cornell voltage and pathological Q waves on the ECG was 1.6%, 7.2%, and 1.8%, respectively.

### Determinants of LVH

**Figure 2:**
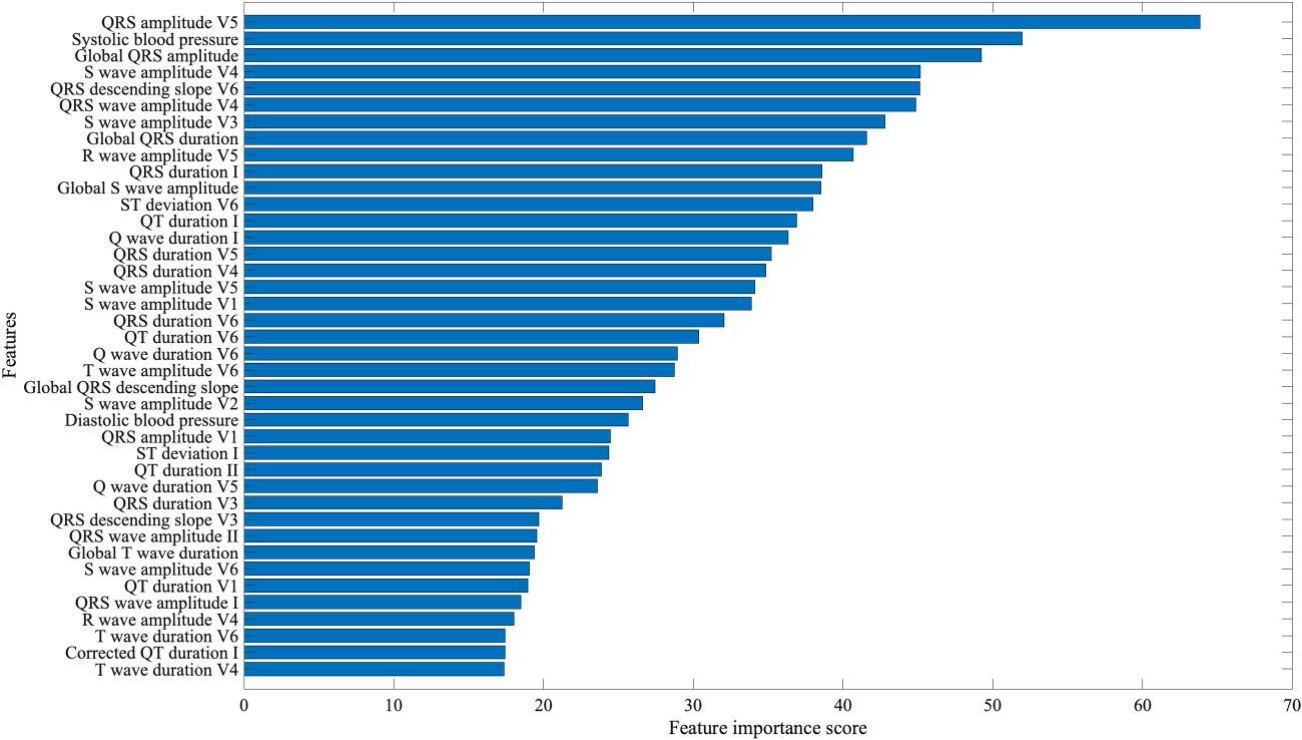
Ranking of the top 40 features using Chi-square feature selection.

Participants with LVH had higher systolic and diastolic BP (*P* < 0.001). Of these, systolic and diastolic BP were also among the top 40 features from chi-squared feature selection. The highest-ranking ECG predictors of LVH were global QRS amplitude and QRS amplitude in V5 (*Figure 2*).

### Machine learning model performance

Overall, the three supervised machine learning models were comparable in classifying LVH. Classification of LVH with logistic regression had an accuracy of 81% (sensitivity 70%, specificity 81%, AUC 0.86), SVM 81% accuracy (sensitivity 72%, specificity 81%, AUC 0.85), and RF 72% accuracy (sensitivity 74%, specificity 72%, AUC 0.83). ECG biomarkers enhanced model performance of all classifiers compared to using clinical variables alone, for example for SVM AUC was 0.85 using both ECG and clinical variables and 0.65 using only clinical data (*Table 2*). *Table 3* shows the validation testing using the UK Biobank imaging centres in rotation for training and test sets including the proportion of participants with LVH. It showed that the three validation tests had similar performance metrics with 0.87, 0.85, and 0.86 AUC values.

**Table 2:** Performance metrics of supervised machine learning classifiers using clinical features only to using both ECG and clinical features

	Logistic Regression		Support Vector Machine		Random Forest	
	Clinical	ECG + clinical	Clinical	ECG + clinical	Clinical	ECG + clinical
Accuracy (%)	69	81	55	81	64	72
Sensitivity (%)	64	70	66	72	57	74
Specificity (%)	69	81	55	81	64	72
AUC	0.71	0.86	0.65	0.85	0.64	0.83

AUC: area under the receiver operator curve, ECG: electrocardiogram.

**Table 3:** Validation by UK Biobank imaging centre using support vector machine

	Validation 1	Validation 2	Validation 3
Training set (Participants with LVH)	Cheadle and Newcastle (513)	Newcastle, Reading and Bristol (166)	Cheadle, Reading and Bristol (477)
Test set (Participants with LVH)	Reading and Bristol (65)	Cheadle (412)	Newcastle (101)
Accuracy (%)	83	81	84
Sensitivity (%)	80	72	74
Specificity (%)	83	81	84
AUC	0.87	0.85	0.86

AUC: area under the receiver operator curve, LVH: left ventricular hypertrophy.

## Discussion

### Summary of findings

In this large, prospective population study, we found that a combination of ECG biomarkers and clinical variables were able to discriminate between participants with normal LV mass and LVH defined by CMR imaging (*Table 2*). We found that the three supervised machine learning classifiers had similar performance in discriminating LVH from normal LV mass. We also demonstrated the incremental value of using the 12-lead ECG compared to clinical variables alone for LVH detection. Validation testing using a rotation of the UK Biobank imaging centres demonstrated robustness of our models with reproducibility of AUC values at different sites (*Table 3*).

### ECG and clinical predictors of LVH

QRS amplitude and interval-based indices were chosen in the feature selection step as being the best classifiers. It is common knowledge that changes in the QRS complex is a marker of electrical remodelling seen in LVH. This is due to the increase in the muscle mass of the LV increasing the forces of the LV potential. However, the increased QRS voltage is seen only in a minority of LVH cases in both clinical and animal studies and consequently voltage criteria suffer from a high number of false negative results and low sensitivity.^35^ The classical LVH criteria such as Sokolow–Lyon, Cornell voltage, and QT dispersion did not appear in the top 40 features. Systolic and diastolic BP were in the top 40 ranking clinical predictors during the feature selection process. This is perhaps expected given that hypertension is the commonest cause of LVH. Despite this, the addition of ECG biomarkers improved model performance of all three classifiers compared to using clinical variables alone. Historically, ECG predictors of LVH have suffered low sensitivity, ranging 15–30%. Using a combination of ECG and clinical variables our sensitivity values were at least 70% without compromising on specificity.

### Comparison of supervised machine learning techniques

The UK Biobank cohort is a relatively healthy, homogenous population, hence the low prevalence of LVH. Class imbalance is a common challenge in machine learning, with different techniques proposed to address this issue. Imbalanced datasets degrade the performance of the classifier with the overall accuracy biased to the majority class.^36^ We applied down-sampling in the training set to minimise this risk. Overall, the three supervised machine learning classifiers were equivalent in performance metrics.

SVMs were initially proposed by Boser, Guyon, and Vapnik in 1992.^37^ In practical classification tasks, logistic regression and linear SVMs often yield very similar results. Logistic regression tries to maximize the conditional likelihoods of the training data, which makes it more prone to outliers than SVMs, which mostly prioritises the points that are closest to the decision boundary (support vectors). On the other hand, logistic regression has the advantage that it is a simpler model and can be implemented more easily. Furthermore, logistic regression models can be easily updated, which is appealing when working with streaming data.^38^ RF is a machine learning algorithm initially introduced by Breiman in 2001.^39^ RF is a classification algorithm using an ensemble of decision trees and its main advantage over SVM is that its less computationally intensive therefore take less time to construct. However, SVMs generally have a higher classification accuracy than RF models as also demonstrated in our experiments.^40^

### Comparison with contemporary research

In our study, we selected ECG features with known physiological association with LVH, hence the supervised machine learning approach. A contemporary study has used deep learning to explore the discriminative power of ECG indices in LVH. Khurshid and colleagues (2021) developed a deep learning model to predict CMR derived LV mass using 12-lead ECG from the UK Biobank cohort.^10^ Khurshid *et al*. used ‘concordance’ statistic or c-statistic to measure model performance, which is comparable to AUC. The authors reported a c-statistic of 0.63 using the deep learning model to predict LV mass. In our study, we demonstrated an AUC of 0.83–0.86. However, we aimed to classify LVH based on a binary classification of normal LV mass vs. LVH, whereas Khurshid and colleagues aimed to estimate the CMR derived LV mass using a regression model, therefore the outcome measure is not comparable. In addition, our supervised machine learning approach also informs about the features contributing to LVH.

### Clinical utility

Machine learning models based on ECG predictors offer new opportunities for improved and cost-effective disease detection, therefore enhancing capabilities of non-specialists. As Angelaki and colleagues (2021) demonstrated, machine learning techniques can be used to predict subclinical disease and therefore has the potential to be used for disease testing, assessing disease progression and thus advance personalised medicine at a lower cost.^41^ This will optimise the use of cardiovascular imaging, ensuring that advanced imaging tests are used for those who need it most, therefore reducing unnecessary testing. Cost-effective and accurate risk prediction of LVH may facilitate population screening and timely treatment in individuals with subclinical disease and could serve as surrogate markers for predicting outcomes. In our study we extracted biomarkers from the 12-lead resting ECG, known to have a physiological association with LVH. As data from the use of wearable devices increases, this offers opportunities to explore ECG biomarkers derived from smartwatches as these become more commonplace. Our study is a first step to explore how well supervised machine learning algorithms work and any implementation of these models for clinical utility would potentially be down the line following independent validation and cost-benefit analyses.

### Strengths and limitations

A strength of our study is the population size, and the UK Biobank imaging study using CMR, as this is the gold standard approach to LVH imaging diagnosis. Access to resting 12-lead ECG of each participant allowed extraction of a number of different ECG biomarkers with a known association with LVH. There are a number of LVH classification systems which are disease specific, most commonly for aortic stenosis and hypertension.^42,43^ We decided upon a binary classification approach to initially predict LVH diagnosis before exploring disease specific cases. In addition to Sokolow–Lyon and Cornell voltage, there are also other ECG criteria proposed for LVH detection such as the Romhilt–Estes score.^44^ However, we focused on the more commonly used scoring systems and, furthermore, the numerous ECG markers we have extracted include many of the components of the aforementioned criteria. The UK Biobank population has low prevalence of LVH. Although we have shown robustness of our algorithm with validation using a rotation of the UK Biobank imaging centres, the next step would be to test our models in an independent dataset for external validation to assess model performance in a population with a higher prevalence of LVH. Another important limitation is that the UK Biobank cohort is predominantly White European, therefore our findings cannot be generalized to other ethnicities, reemphasising the need for external validation. The goal of this study was to predict LVH as a binary variable. We have included the prevalence of the more common causes of LVH such as hypertension and hypertrophic cardiomyopathy (*Table 1*) but there are also rarer causes such as infiltrative conditions which have not been fully classified in the UK Biobank cohort.

## Conclusions

A combination of automatically extracted ECG biomarkers and clinical variables were able to classify LVH defined by CMR. Our findings provide support for the ECG as an inexpensive screening tool to risk stratify patient with LVH, thereby acting as a gatekeeper to advanced imaging.

## Supplementary Material

ztad037_Supplementary_Data

## Data Availability

The data underlying this article were provided by the UK Biobank under access application 2964. UK Biobank will make the data available to bona fide researchers for all types of health-related research that is in the public interest, without preferential or exclusive access for any persons. All researchers will be subject to the same application process and approval criteria as specified by UK Biobank. For more details on the access procedure, see the UK Biobank website: http://www.ukbiobank.ac.uk/register-apply/.
