# Supplementary material for: Predicting left ventricular hypertrophy from the 12-lead electrocardiogram in the UK Biobank imaging study using machine learning
Source: Eur Heart J Digit Health. 2023 Jun 1;4(4):316–24. doi: 10.1093/ehjdh/ztad037 (PMC10393938; doi:10.1093/ehjdh/ztad037)
Supplement: ztad037_Supplementary_Data [file ztad037_supplementary_data.docx]

**Supplementary material**

**Figure 1. ECG waveform from precordial lead V6 demarcating QRS complex**


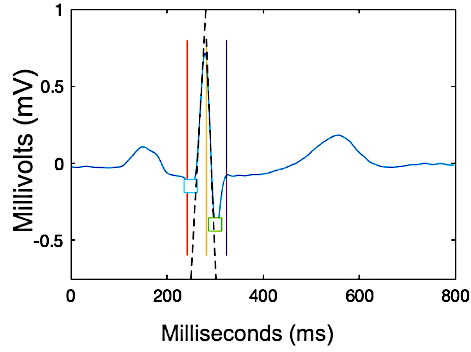


**Legend:** QRS onset (orange vertical line), QRS offset (violet vertical line) and QRS ascending and descending slopes (dotted lines)

**Table 1. ECG biomarkers used in the model**

| **ECG marker** | **Definition** |
| --- | --- |
| Sokolow-Lyon index (mm) | (SV1 or SV2) + (RV5 or RV6) ≥35mm or R wave in aVL ≥11mm |
| Cornell voltage (mm) | SV3 +RaVL ≥28mm (men) ≥20mm (women) |
| Pathological Q waves | >30ms in duration and >1/3 of the R wave in depth in two or more contiguous leads (I, II, V1-6) |
| ST segment deviation (mm) | Amplitude from QRS offset to T onset in relation to the isoelectric line. |
| QT dispersion (ms) | Inter-lead QT variation in QT segment length |
| Corrected QT duration (ms) | QT duration was computed as the difference between Q onset and T wave end and the corrected QT interval was calculated using the Bazett’s formula. |
| Positive deflection P wave amplitude (mV) | Amplitude of P wave from Q onset |
| Positive deflection P duration (ms) | Time interval between p wave offset and onset |
| Negative terminal P wave amplitude (mV) | Amplitude of the terminal negative component of the P-wave |
| Negative terminal P duration (ms) | Interval of the terminal negative component of the P-wave |
| P wave terminal force in V1 | Product of the amplitude of the terminal negative component of the P-wave in V1 (p prime) and the duration (p prime duration) |
| P wave duration interval | Time interval between P onset and offset |
| Q wave amplitude (mV) | Amplitude of Q wave from Q onset |
| Q wave duration (ms) | Difference between Q wave onset and offset |
| R wave amplitude (mV) | Amplitude of R wave from Q onset |
| S wave amplitude (mV) | Amplitude of S wave from Q onset |
| QRS amplitude (mV) | Difference between the maximum and minimum points of the QRS complex |
| QRS duration (ms) | Time interval between QRS offset and QRS onset. |
| QRS ascending slope (mV) | Maximum slope between QRS onset and QRS peak |
| QRS descending slope (mV) | Minimum slope from QRS peak to QRS offset |
| T wave amplitude (mV) | Maximal amplitude following the QRS complex |
| T wave duration (ms) | Difference between T wave onset and T wave end |
